# Supplementary figures and images for: Generation of Chimeric African Swine Fever Viruses Through In Vitro and In Vivo Intergenotypic Gene Complementation
Source: Vaccines (Basel). 2025 Apr 25;13(5):462. doi: 10.3390/vaccines13050462 (PMC12115777; doi:10.3390/vaccines13050462)

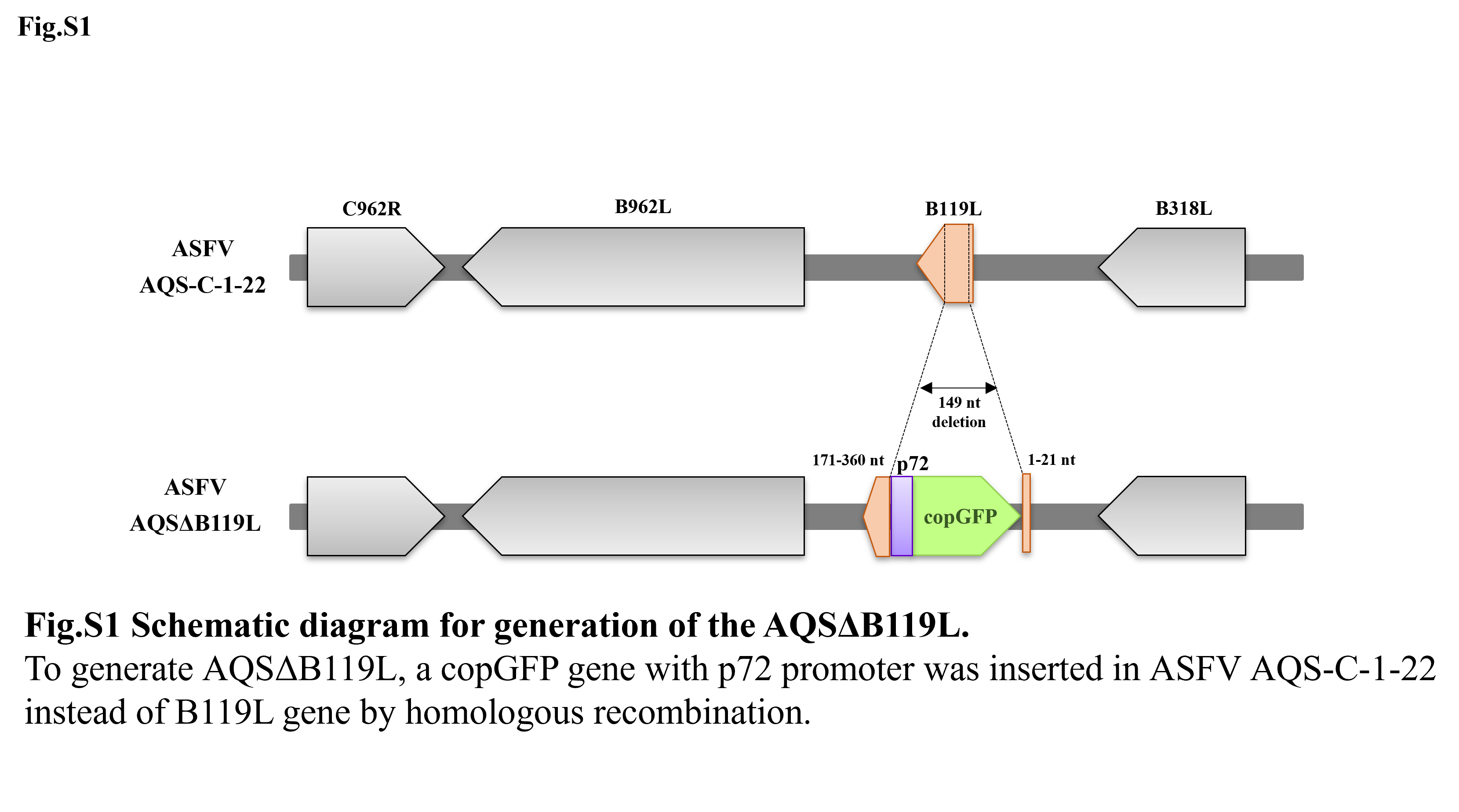

Supplement: Supplementary file 1 [file vaccines-13-00462-s001.zip › FigS1.png]

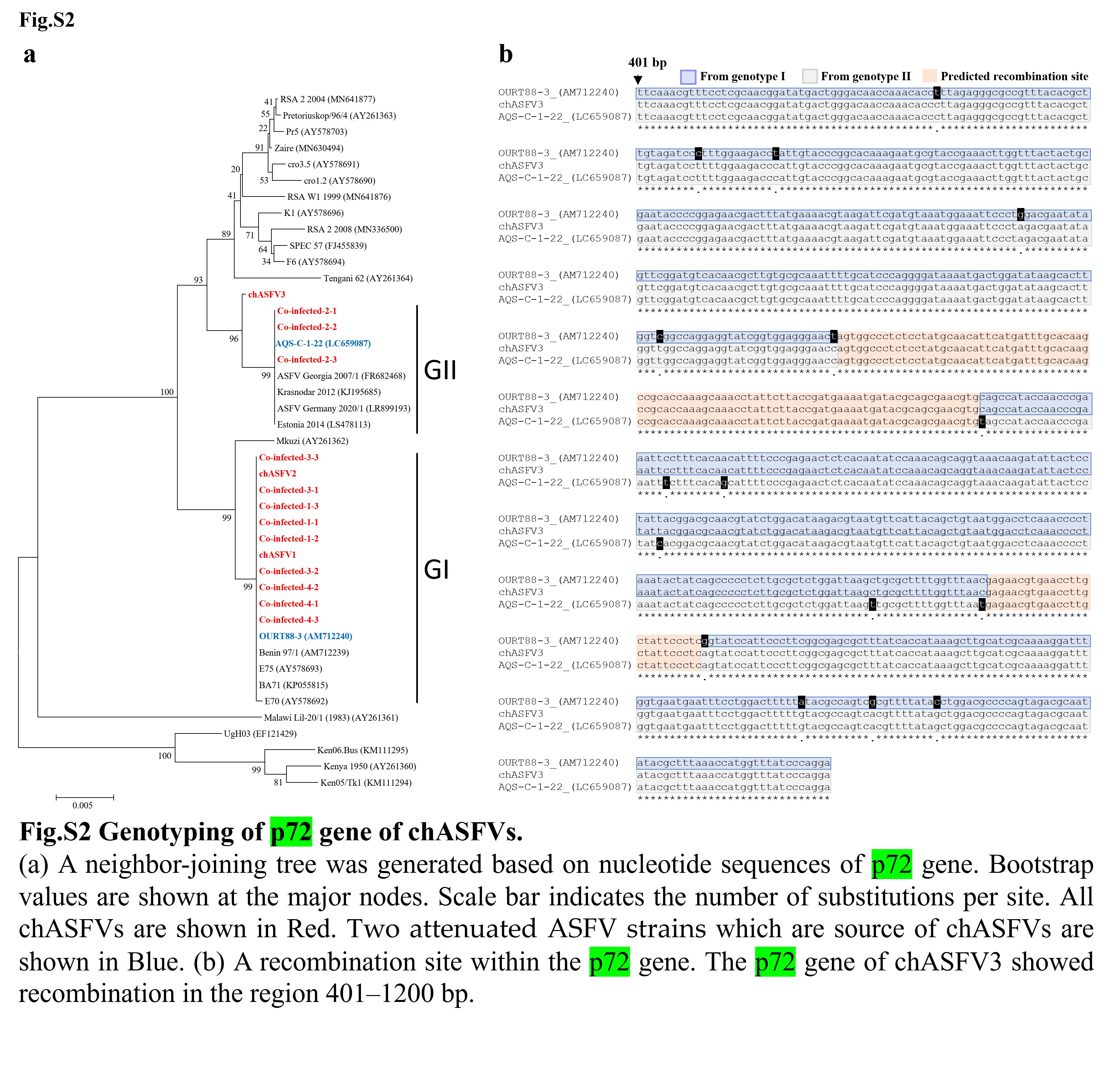

Supplement: Supplementary file 1 [file vaccines-13-00462-s001.zip › Revised FigS2.jpg]
